# A giant head and neck hemangioma of the fetus: A case report

**DOI:** 10.1097/MD.0000000000035855

**Published:** 2023-11-24

**Authors:** Xueying Wang, Hongwei Zhang, Maochun Zhang, Yuanyuan Guo

**Affiliations:** a Department of Ultrasound, Affiliated Hospital of North Sichuan Medical College, Nanchong, Sichuan Province, China; b Department of Obstetrics and Gynecology, Affiliated Hospital of North Sichuan Medical College, Nanchong, Sichuan Province, China.

**Keywords:** case report, fetal edema, heart failure, hemangioma, ultrasound

## Abstract

**Rationale:**

Hemangioma is a common benign disease in clinical practice, but it is rare to find a giant hemangioma in the fetal period.

**Patient concerns:**

Here, we report a case of a giant hemangioma of the fetal head and neck measuring approximately 10.1 × 6.5 cm.

**Diagnoses:**

At first, only ultrasonography was used to diagnose the suspected hemangioma. The pregnant woman refused to undergo further testing and requested induction of labor, after which the tumor was finally sent for pathological examination to confirm hemangioma.

**Interventions and outcomes:**

Additionally, the fetus developed severe edema (fluid accumulation in the thoracic, abdominal, and pericardial cavities), which can be fatal to the fetus. Finally, the mother refused to continue the pregnancy and underwent induction of labor with rivanol.

**Lessons:**

Most hemangiomas are small and asymptomatic. Giant hemangiomas are rare and associated with a variety of maternal and fetal complications. Therefore, this article aims to summarize the knowledge related to hemangioma through this case, strengthen doctors’ understanding of this disease, and bring the attention of pregnant women to this disease to ensure early diagnosis and treatment and prevent a poor prognosis.

## 1. Introduction

Hemangiomas can occur in many parts of the body, including the head and neck, face, viscera, limbs, and skin. It is rare to find a giant hemangioma in a fetus. Hemangioma may be related to chromosome abnormalities, maternal inheritance, placental origin, etc.^[1]^ The ultrasonic manifestations of hemangioma are mainly mixed solid/cystic echo or solid echo. Most small hemangiomas resolve spontaneously after birth, without treatment, and the prognosis of the fetus is good.^[2]^ However, fetal hemangioma may grow gradually during the whole pregnancy, and fetuses with large hemangiomas are prone to high output heart failure, platelet aggregation and other complications, resulting in an extremely poor prognosis.^[3]^ Here, we describe a rare case of fetal giant hemangioma based on the ultrasound image changes, which was confirmed by pathology.

## 2. Case presentation

**Figure 1.**
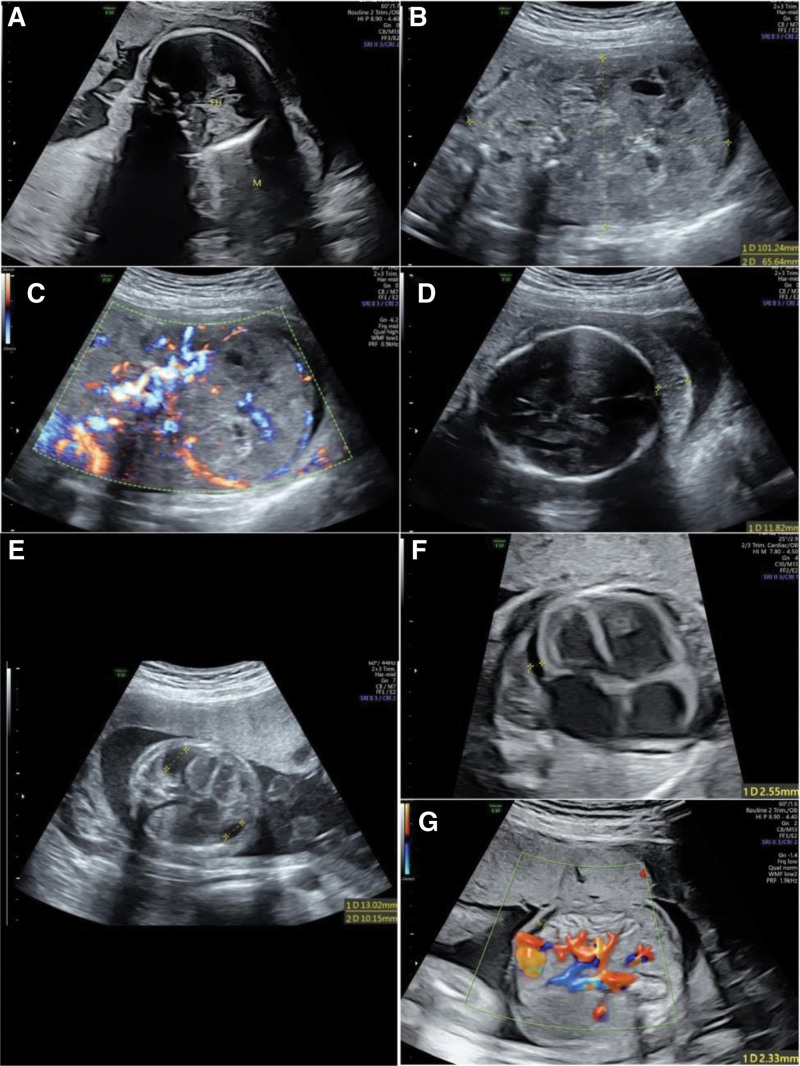
Targeted ultrasound sonogram. (A) Sagittal view of the mass in the head and neck. FH = fetal head, M = mass. (A–C) A heterogeneous solid mass measuring approximately 10.1 × 6.5 cm was observed in the head and neck, and rich blood flow signals were observed in the mass. (D) Fetal scalp edema, measuring approximately 1.1 cm wide. (E) Pleural effusion. The width of the left side measured approximately 1.3 cm and the width of the right side measured approximately 1.0 cm. (F) Pericardial effusion. It measured approximately 0.3 cm wide. (G) Fluid in the abdominal cavity. It measured approximately 0.2 cm wide.

A 35-year-old pregnant woman presented to her local hospital and was found to have an abnormal fetus at 23^+6^ weeks of gestation. There was an abnormally strong echo between the scalp and the skull, and the fetus had partial scalp thickening and an enlarged heart. Before this, the pregnant woman had received regular prenatal examinations, with no obvious abnormalities. Two days later, the pregnant woman came to see us in our hospital. We performed a targeted ultrasound examination of the fetus during pregnancy (Fig. 1) and found a large lump present in the fetus’s head and neck and fetal scalp edema. We also observed that the fetal chest, abdominal cavity and pericardial cavity had a small amount of effusion. We obtained the patient’s verbal and written informed consent before publishing the case.

**Figure 2.**
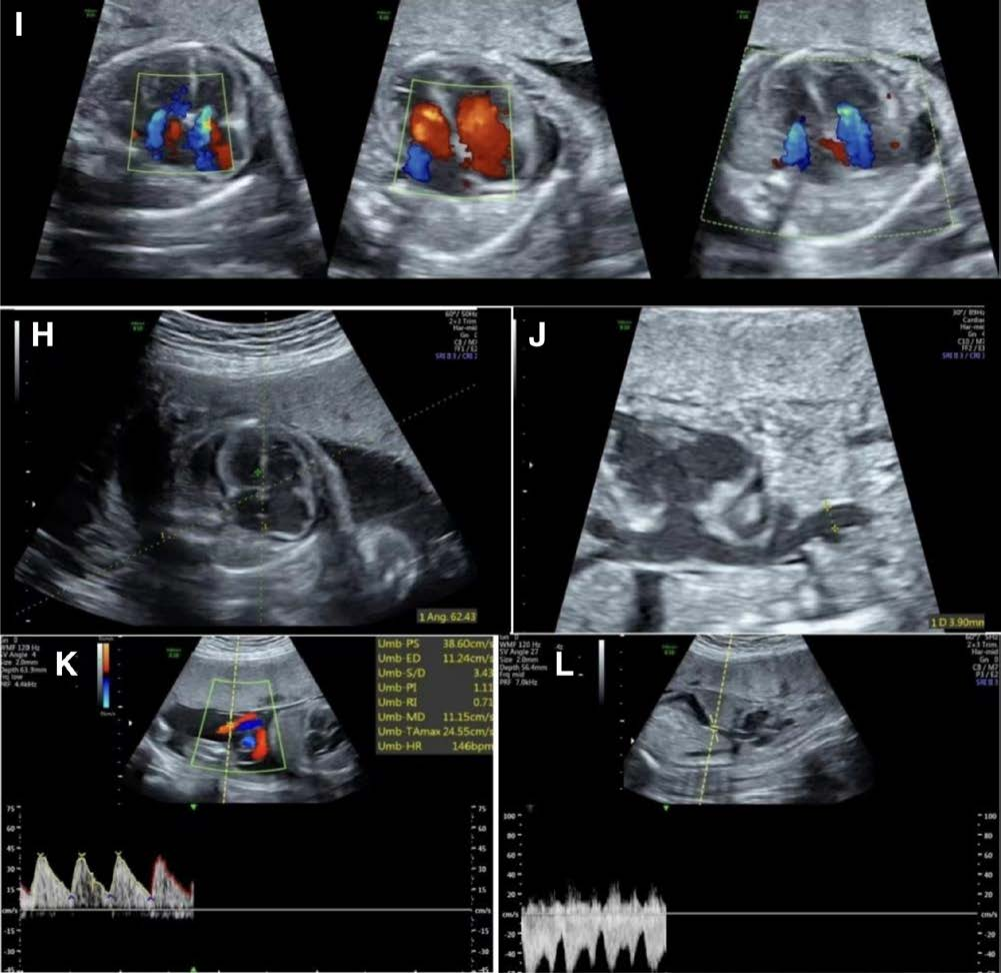
Fetal echocardiography. (H) The fetal heart was enlarged and the cardiothoracic ratio was more than 1/2. (I) There was reverse flow signal in both mitral and tricuspid valves. (J) The superior vena cava measured approximately 0.39 cm wide. (K and L) The umbilical artery and venous catheter spectrum of the fetus were not abnormal.

From the fetal echocardiogram (Fig. 2), we found that the fetal heart was enlarged, the cardiothoracic ratio was more than 1/2, and there was a moderate amount of reverse flow signal in both the mitral and tricuspid valves. However, the umbilical artery and venous catheter spectrum of the fetus were normal, and the rest of the images were normal. Last, the fetal cardiovascular profile score was 4.

**Figure 3.**
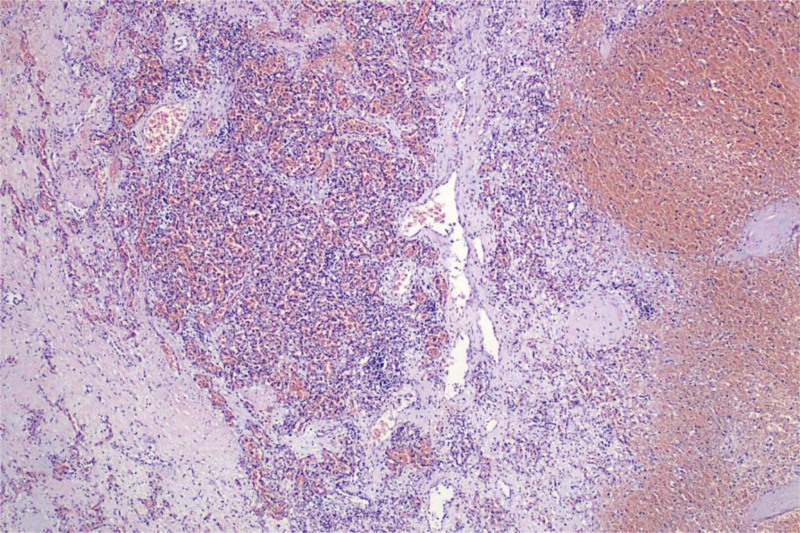
Pathology of fetal head and neck mass, H&E staining (×40). Microscopically, the mass shows a large number of red blood cells, consistent with hemangioma.

Then, from the overall ultrasonography, we considered that the huge mass in the head and neck of the fetus might be hemangioma. The tumor was large, there was vascular anastomosis in the tumor, and the excessive blood circulation caused extreme cardiac load, which resulted in fetal heart failure and fetal edema. Afterward, the pregnant woman and her family refused other tests, such as an magnetic resonance imaging, and received genetic counseling from a specialist doctor. The doctor explained to them that the fetus had a poor prognosis. After careful consideration, they ultimately refused to continue the pregnancy and requested abortion. After all the preparations for surgery, the pregnant woman underwent amniocentesis. After a day of close monitoring, a stillborn fetus was delivered through the vagina. Clinicians removed the head and neck mass of the fetus as a specimen and sent it for pathological examination (Fig. 3), which proved that the head and neck mass was a hemangioma. The patient has been followed up to this day and is currently in good health.

## 3. Discussion

Hemangiomas are common in clinical practice, but giant fetal hemangiomas are not common. The characteristics of hemangiomas in the head and neck are obvious compared with those in other parts and can cause adverse effects on the appearance of the fetus and may affect the physical and mental health of the fetus.^[4]^ Compared with fetuses with small hemangiomas, fetuses with large hemangiomas are more likely to develop high output heart failure complications.^[5]^ However, most cases of small hemangiomas can be resolved after birth.^[6]^ The prognosis of the two fetuses is different. The case we reported was a rare giant fetal head and neck hemangioma, the fetus had developed heart failure and edema, and the prognosis of fetal edema was poor.^[7]^ Finally, the pregnant woman decided to discontinue the pregnancy and underwent rivanol-induced labor.

In addition, fetal head and neck hemangioma also needs to be clarified from other fetal neck masses. Fetal head and neck masses, including neck hydrocystoma, cervical teratoma, fetal goiter, encephalocele, and meningocele, are rare in clinical practice, but the most common is neck hydrocystoma.^[8–10]^ Hydrocystoma is often associated with chromosomal abnormalities, and the typical ultrasound image changes to paracervical or retrocervical cystic masses, which are mostly multilocular with fine compartments and can be associated with severe fetal edema.^[11]^ Although the accompanying symptoms of edema were also present in our case, the mass was heterogeneous and solid in nature, which was different from a cystic tumor, so we did not consider a cystic tumor. Cervical teratoma is a rare tumor because its composition is complicated, with more teeth, bone, fat, and hair. Ultrasonographic changes have different presentations, including solid and cystic echoes or mixed masses. Internal masses occasionally have a star, punctiform blood flow signals, and a lump that can have different sizes of calcification, characterized by a strong echo mass associated with acoustic shadow behind. At the same time, the tumor can compress the esophagus and affect fetal amniotic fluid swallowing resulting in polyhydramnios,^[12]^ but this case did not have such ultrasonographic findings (the amniotic fluid index was approximately 130 mm). In the transverse section of the neck, the fetal goiter can show a symmetrical hypoechoic substantial mass in the anterior area of the neck, connected by an isthmus in the middle, and a circular anechoic trachea behind the isthmus. An encephalocele appears as an echo interruption of the cranial echo ring on ultrasound. The meninges or brain tissue bulge from the location of the echo interruption (i.e., the defect). If the bulge is only composed of meninges, then it is a meningocele. In our case, the skull echo ring was complete, so meningocele was not considered.

It has been suggested that there are two main pathophysiological mechanisms of fetal edema caused by hemangioma. One is that hemangioma manifests as arteriovenous malformations further leading to high output heart failure and to fetal edema. The second mechanism involves hemolytic anemia, similar to Kasabach-Merritt syndrome, resulting in chronic intrauterine hypoxia and fetal growth restriction.^[13]^ At present, a few scholars at home and abroad have tried intrauterine treatment of fetal hemangioma. For example, Quintero et al^[14]^ used fetal endoscopic suturing of tumor blood vessels to treat hemangioma complicated by fetal edema at 24 weeks, but fetal death occurred on the third day after the operation. Bhide et al^[15]^ performed ultrasound-guided laser local treatment for the hemangioma with continuous hyperdynamic circulation, and the prognosis was good. Lau et al^[16]^ used microcoil embolization to treat a hemangioma that was complicated by severe anemia and a hemangioma that was complicated by severe fetal edema, but the two attempts were not successful. There were also reports of the failure^[17]^ and success^[18]^ of ultrasound-guided intravenous injection of absolute alcohol into tumors. Although these methods can make the tumor ischemic or necrotic and make the tumor shrink and disappear, because of its high risk and difficulty, it is not commonly used. At present, radiofrequency ablation is the most commonly reported approach as an alternative treatment for hemangioma, especially hepatic hemangioma.^[19–21]^ Recently, the first successful case of ultrasound-guided intrauterine biopsy and radiofrequency ablation for fetal posterior cervical solid tumor was reported, giving a new approach to the treatment of hemangioma.^[22]^ Now the more mature method is to treat the complications. Amniocentesis releases amniotic fluid when there is excessive amniotic fluid, which can reduce the pressure in the official cavity and relieve the symptoms of pregnant women. Jones et al^[23]^ indicated that the release of amniotic fluid should be treated with caution because it can lead to a decrease in uterine pressure and increase blood perfusion to the tumor, thereby worsening the fetal condition. The indications for drainage were an amniotic fluid index ≥ 40 cm or a maximum amniotic fluid depth ≥ 12 cm or obvious symptoms of maternal compression.^[17]^ Intrauterine blood transfusion is used when the fetus is anemic, and treatment is carried out before edema; otherwise, the effect is not good, and there are certain risks and difficulties.

As an important prenatal diagnostic method, and compared with magnetic resonance imaging, ultrasound is simple, inexpensive and widely used in clinical practice. This case is a good example of adverse complications and poor prognosis of large hemangioma. Due to complications such as heart failure and edema when the hemangioma was detected by ultrasound, the best opportunity for early intrauterine treatment for the fetus was missed, resulting in a poor outcome. However, for a pregnant woman who received regular prenatal examinations yet had a fetus with a hemangioma that had grown so large in <24 weeks, speculation of whether the diagnosis was missed earlier in her care is inevitable, particularly given that the hemangioma detection rate is not high. Therefore, through this case report and summary, we hope to improve the understanding of hemangioma among clinicians, especially our sonographers, and increase the detection rate of hemangioma. When the prenatal examination finds that the fetus may have a hemangioma, the doctor should pay attention and inform the pregnant woman of its importance. Pregnant women should also be diligent about their own care and be regularly monitored by ultrasound. In addition to monitoring the change in size of the hemangioma, sonographers should also focus on monitoring amniotic fluid volume, fetal development, the cardiothoracic ratio, whether there is edema, various obstetric Doppler data and hemodynamic changes to prevent a poor prognosis.

## Author contributions

**Conceptualization:** Hongwei Zhang, Maochun Zhang, Yuanyuan Guo.

**Data curation:** Xueying Wang.

**Formal analysis:** Xueying Wang.

**Funding acquisition:** Maochun Zhang.

**Investigation:** Xueying Wang, Hongwei Zhang.

**Methodology:** Xueying Wang, Hongwei Zhang.

**Resources:** Xueying Wang, Hongwei Zhang, Maochun Zhang, Yuanyuan Guo.

**Supervision:** Xueying Wang, Maochun Zhang.

**Validation:** Xueying Wang.

**Writing – original draft:** Xueying Wang.

**Writing – review & editing:** Xueying Wang, Hongwei Zhang, Maochun Zhang, Yuanyuan Guo.
